# Supplementary material for: Synthesis and Behavior of DNA Oligomers Containing the Ambiguous Z-Nucleobase 5-Aminoimidazole-4-carboxamide
Source: Molecules. 2023 Apr 6;28(7):3265. doi: 10.3390/molecules28073265 (PMC10096470; doi:10.3390/molecules28073265)
Supplement: Supplementary file 1 [file molecules-28-03265-s001.zip › molecules-2301844-supplementary.pdf]

Synthesis and behavior of DNA oligomers containing the ambiguous Z-nucleobase 5-aminoimidazole-4-carboxamide

Yuhei Nogi[1], Noriko Saito–Tarashima[1],* Sangita Karanjit[1], and Noriaki Minakawa[1,*]

1 Graduate School of Pharmaceutical Science, Tokushima University.

* Correspondence: noriko.tarashima@tokushima-u.ac.jp (N.S.T.); minakawa@tokushima-u.ac.jp (N.M.)

Table of Contents

Page

1. **Figure S1.** Synthesis of the **ODN4** containing multiple Z-bases. S1.
2. **Figure S2.** Proposed mechanism of Z-base construction by ring-opening reaction of an Hxa^{DNP}-base (DFT calculation result with the calculated activation barriers). S2.
3. **Figure S3.** Possible formation of a Hoogsteen-type Z:G pair similar to a natural A:G pair. S3.
4. **Table S1.** Characterization of ODNs containing Z-bases. S4.
5. NMR spectra of compounds **2–4**. S5.
6. Cartesian coordinates of intermediates and transition states S10.

Figure S1. Synthesis of the **ODN4** containing multiple Z-bases. LC-MS analysis after treatment by (a) ethylenediamine at 50 °C, and (b) 1,3-propanediamine at 50 °C. Samples were analyzed by UPLC using a BEH C18 column (Waters, 2.1×50 mm, 1.7 μm) eluted with linear gradient from 0% to 60% MeOH with 3.5 mM TEA and 40 mM HFIP (0.3 mL/min for 15 min).

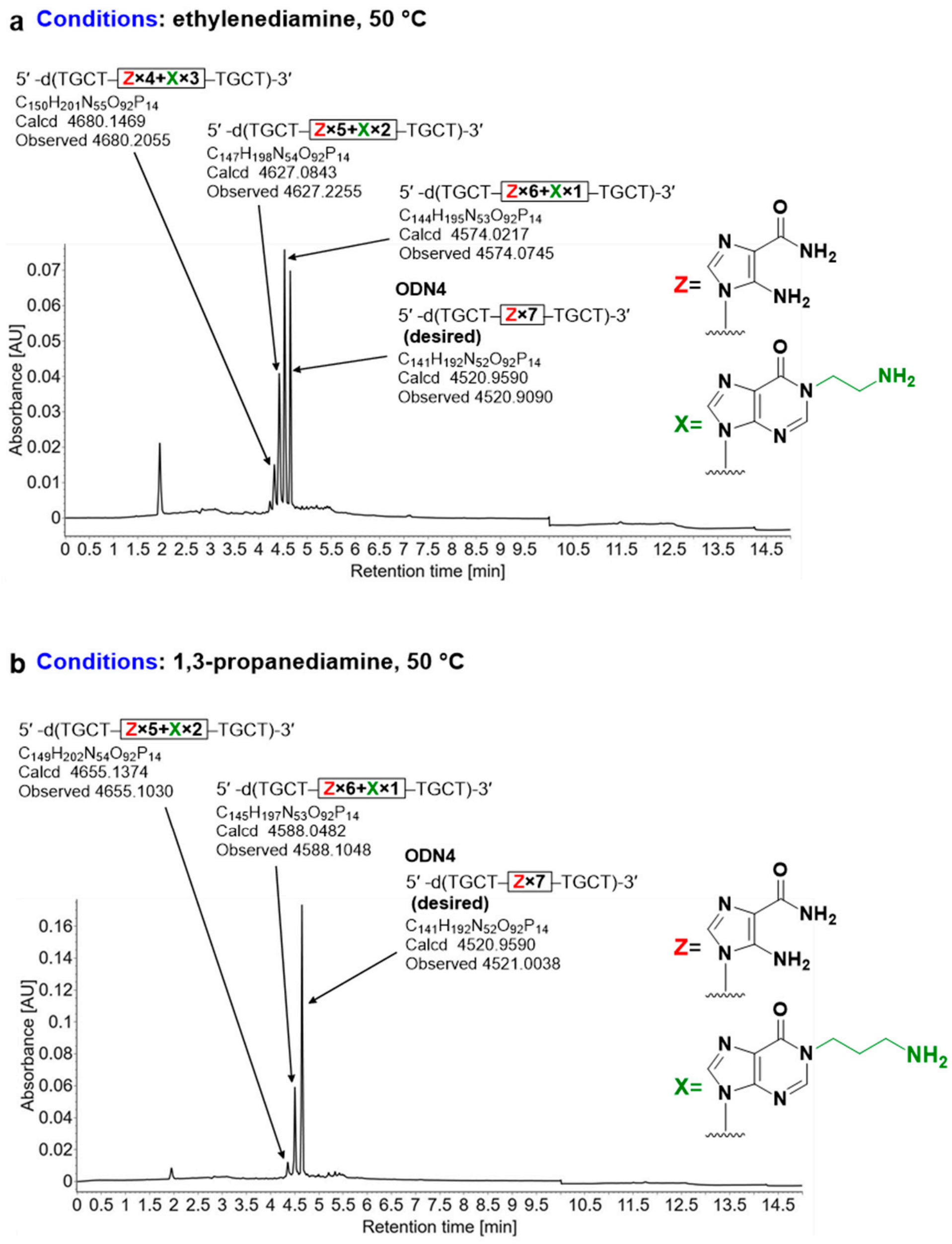

Figure S2. Proposed mechanism of Z-base construction by ring-opening reaction of an Hxa^{DNP}-base (DFT calculation result with the calculated activation barriers).

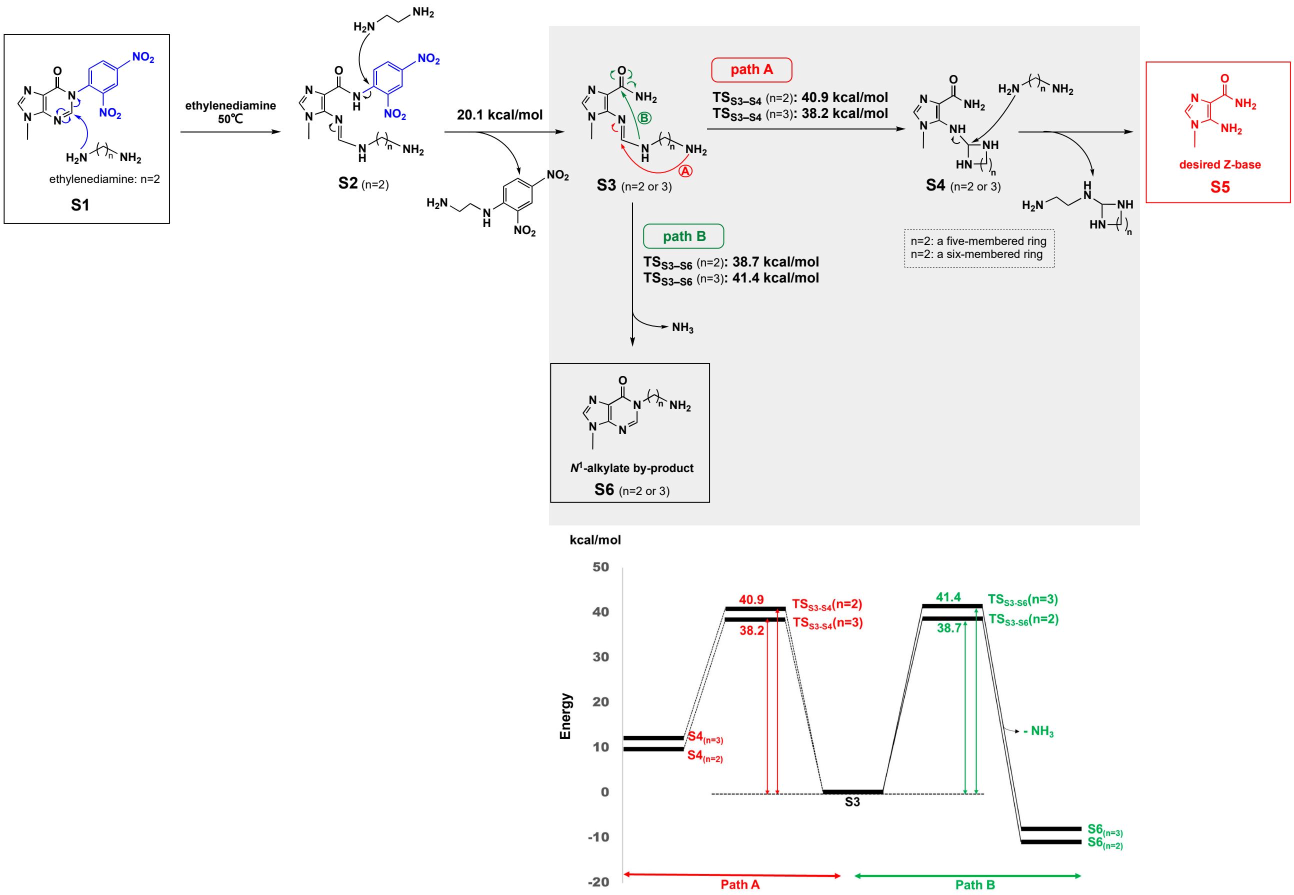

Figure S3. Possible formation of a Hoogsteen-type Z:G pair similar to a natural A:G pair.

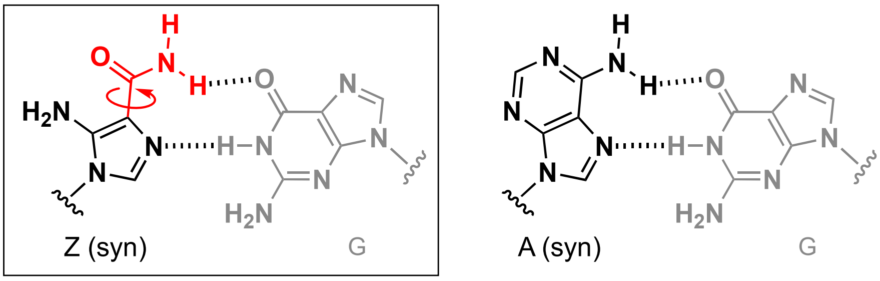

Table S1. Characterization of ODNs containing Z-bases.

#	Squence (5′→3′)	Fomula	Calcd. mass	Observed mass	Correspond duplexes
ODN2	d(TGCTACA**Z**ACATGCT)	$C_{145}H_{186}N_{54}O_{88}P_{14}$	4526.9700	4526.9759 (ethylenediamine) 4526.9641 (1,3-propandiamine)	#1–#4 (Table 1)
ODN4	d(TGCT**ZZZZZZZ**TGCT)	$C_{141}H_{192}N_{52}O_{92}P_{14}$	4520.9590	4521.0038	–
ODN5	d(TGCTACC**Z**CCATGCT)	$C_{143}H_{186}N_{50}O_{90}P_{14}$	4478.9206	4478.9618	#10–#13 (Table 1)
ODN6	d(**Z**CGCGCG)	$C_{66}H_{86}N_{28}O_{40}P_{6}$	2097.3952	2097.3912	#3 (Table 2)
ODN7	d(GTGGGCAAG**Z**GTGCGCTGACCATCCAGAAC)	$C_{291}H_{366}N_{121}O_{175}P_{29}$	9256.9645	9256.9596	Figure 3a
ODN8	d(GGGACTAGCTACGAGTGCTCCCTGTATACTTACTC GTA**Z**GTCGTCGAACTACCACGGCCACTTCAGACCACC AGCTTATATTCCGTC)	$C_{841}H_{1068}N_{312}O_{523}P_{86}$	26578.9987	26578.1173	Figure 3d

NMR spectra

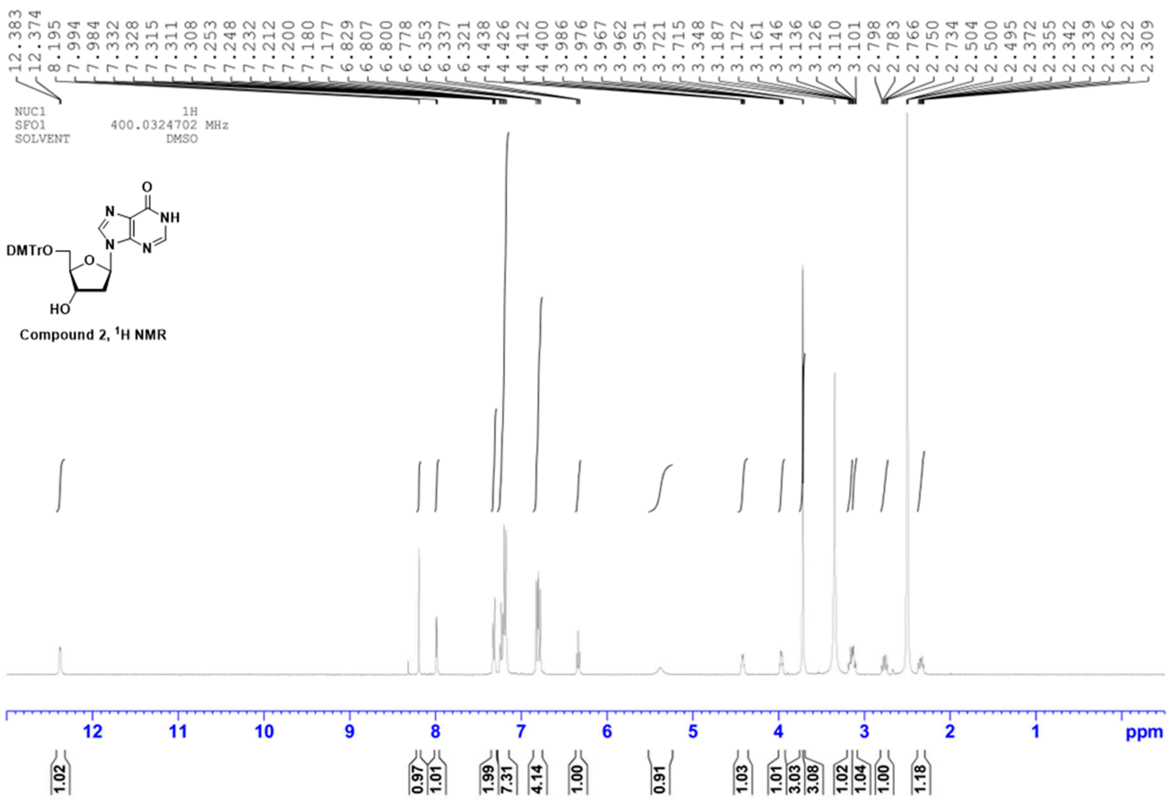

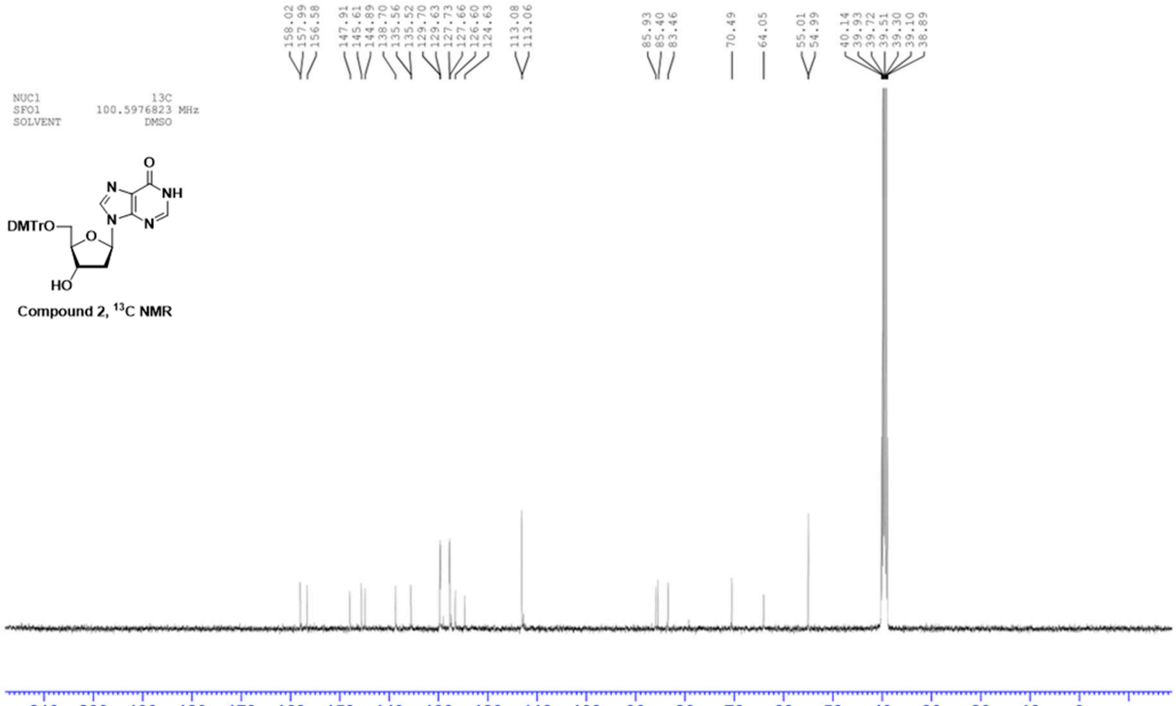

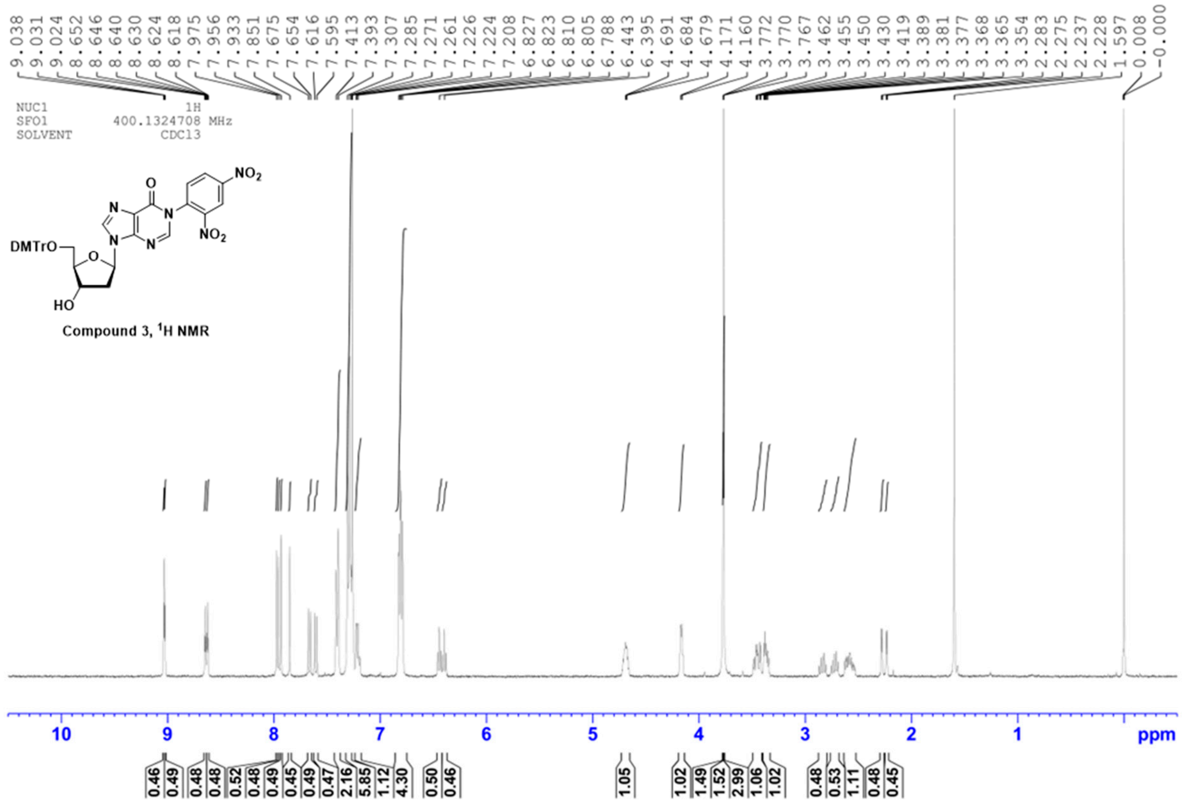

9.038 9.031 9.024 8.652 8.646 8.640 8.630 8.624 8.618 7.975 7.956 7.933 7.851 7.675 7.654 7.616 7.595 7.413 7.393 7.307 7.285 7.271 7.261 7.226 7.224 7.208 6.827 6.823 6.810 6.805 6.788 6.443 6.395 4.691 4.684 4.679 4.171 4.160 3.772 3.770 3.767 3.462 3.455 3.450 3.430 3.419 3.389 3.381 3.377 3.368 3.365 3.354 2.283 2.275 2.237 2.228 1.597 0.008 -0.000
NUC1 1H
SFO1 400.1324708 MHz
SOLVENT CDCl3
NO2
NO2
DMTrO
HO
Compound 3, 1H NMR
10 9 8 7 6 5 4 3 2 1 ppm
0.46 0.49 0.48 0.48 0.52 0.48 0.49 0.45 0.49 0.47 2.16 5.85 1.12 4.30 0.50 0.46 1.05 1.02 1.49 1.52 2.99 1.06 1.02 0.48 0.53 1.11 0.48 0.45

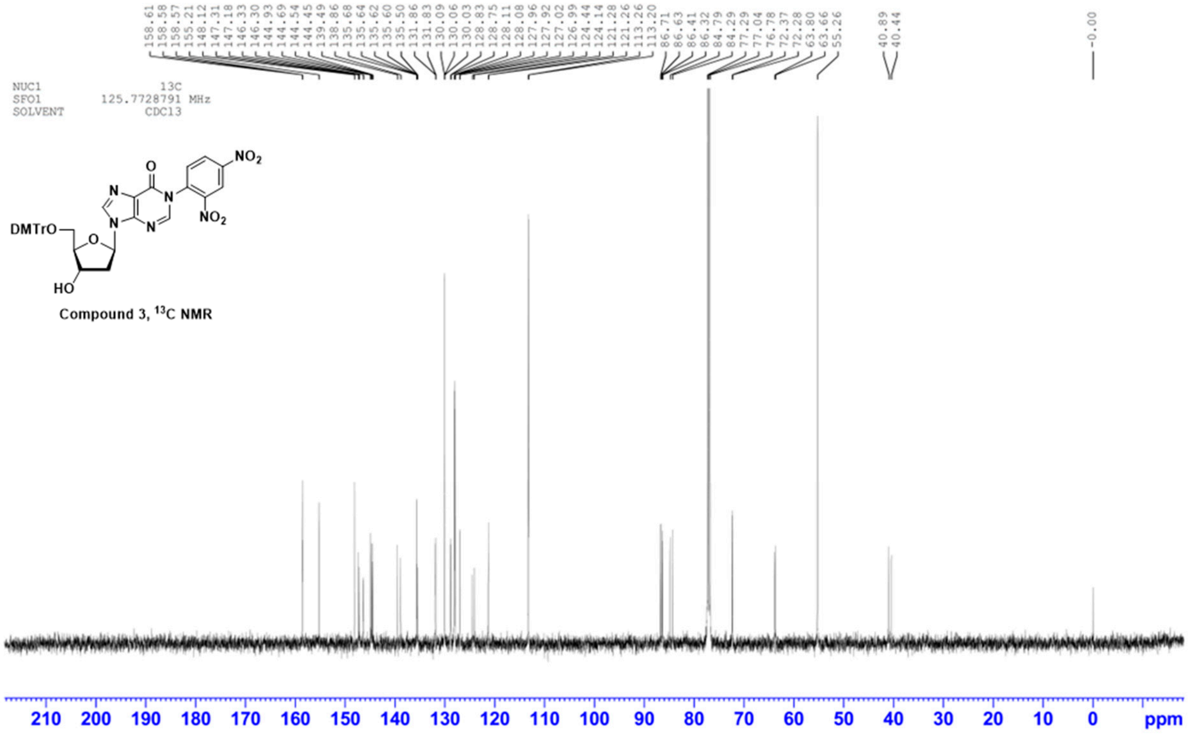

158.61 158.58 158.57 155.21 148.12 147.31 147.18 146.33 146.30 144.93 144.69 144.54 144.45 139.49 138.86 135.68 135.64 135.62 135.60 135.50 131.86 131.83 130.09 130.06 130.03 128.83 128.75 128.11 128.08 127.96 127.92 127.02 126.99 124.44 124.14 121.28 121.26 113.26 113.20 86.71 86.63 86.41 86.32 84.79 84.29 77.29 77.04 76.78 72.37 72.28 63.80 63.66 55.26 40.89 40.44 -0.00
NUC1 13C
SFO1 125.7728791 MHz
SOLVENT CDCl3
NO2
NO2
DMTrO
HO
Compound 3, 13C NMR
210 200 190 180 170 160 150 140 130 120 110 100 90 80 70 60 50 40 30 20 10 0 ppm

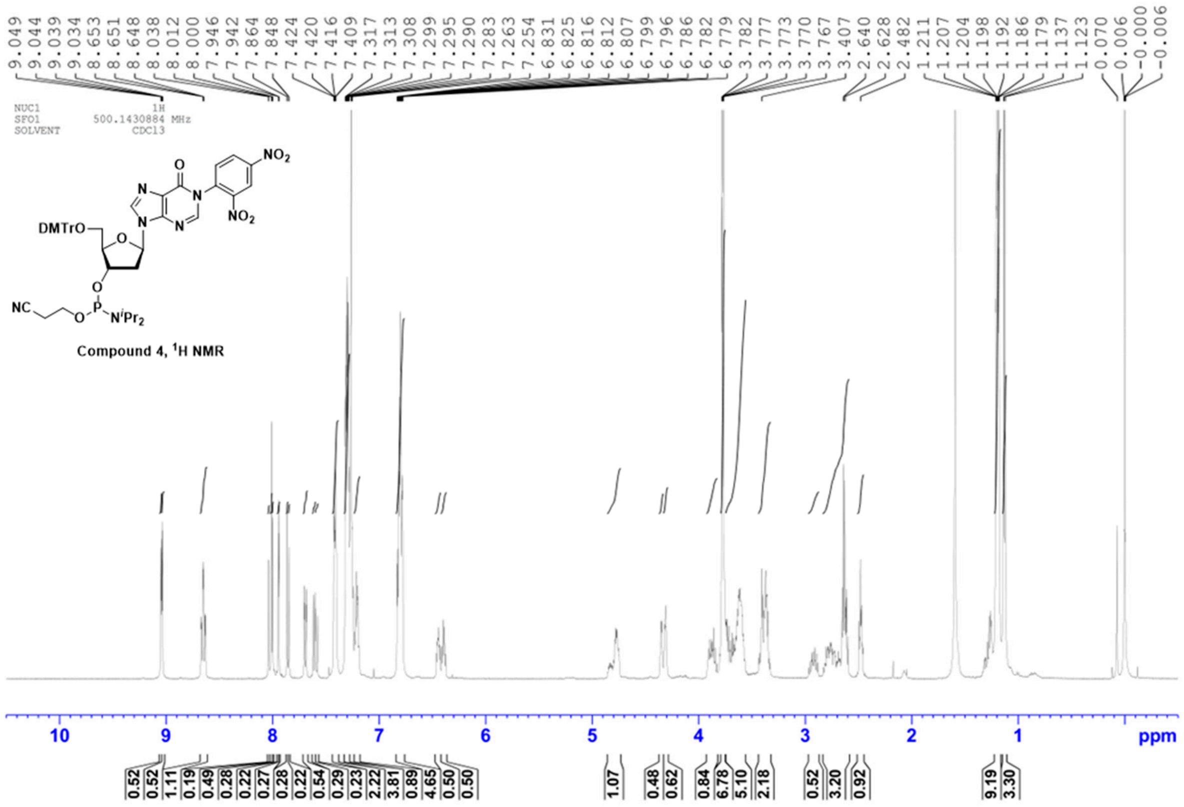

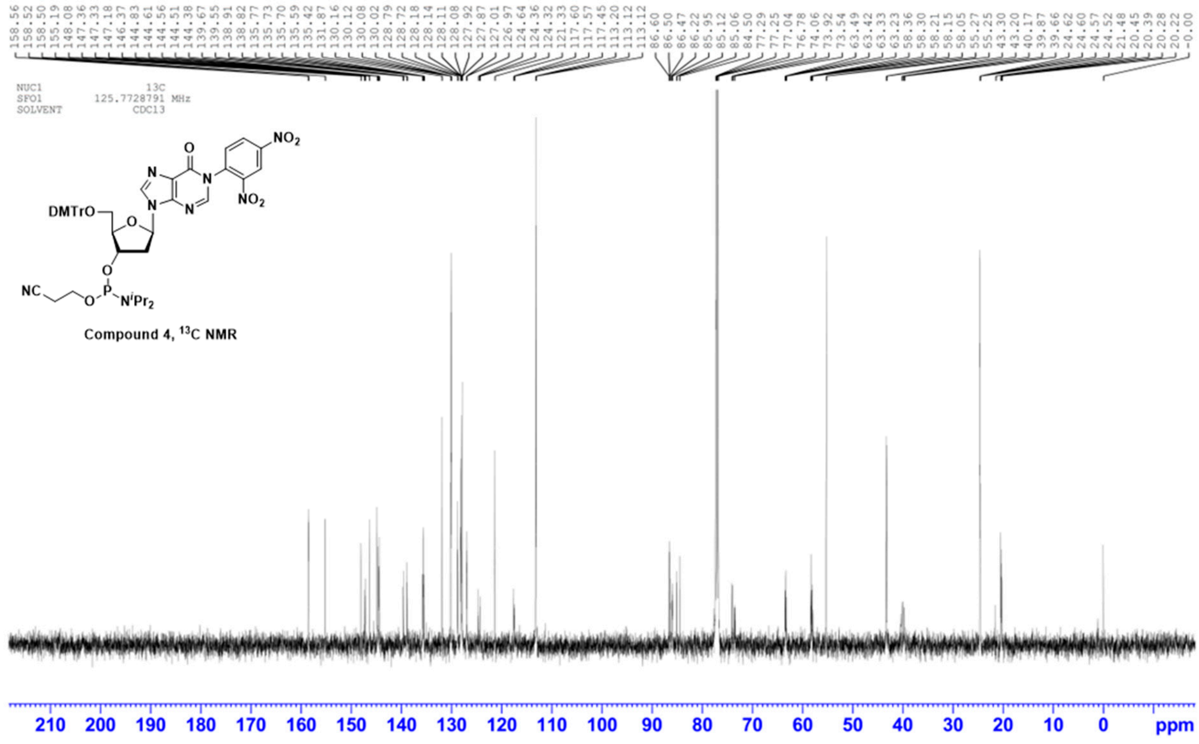

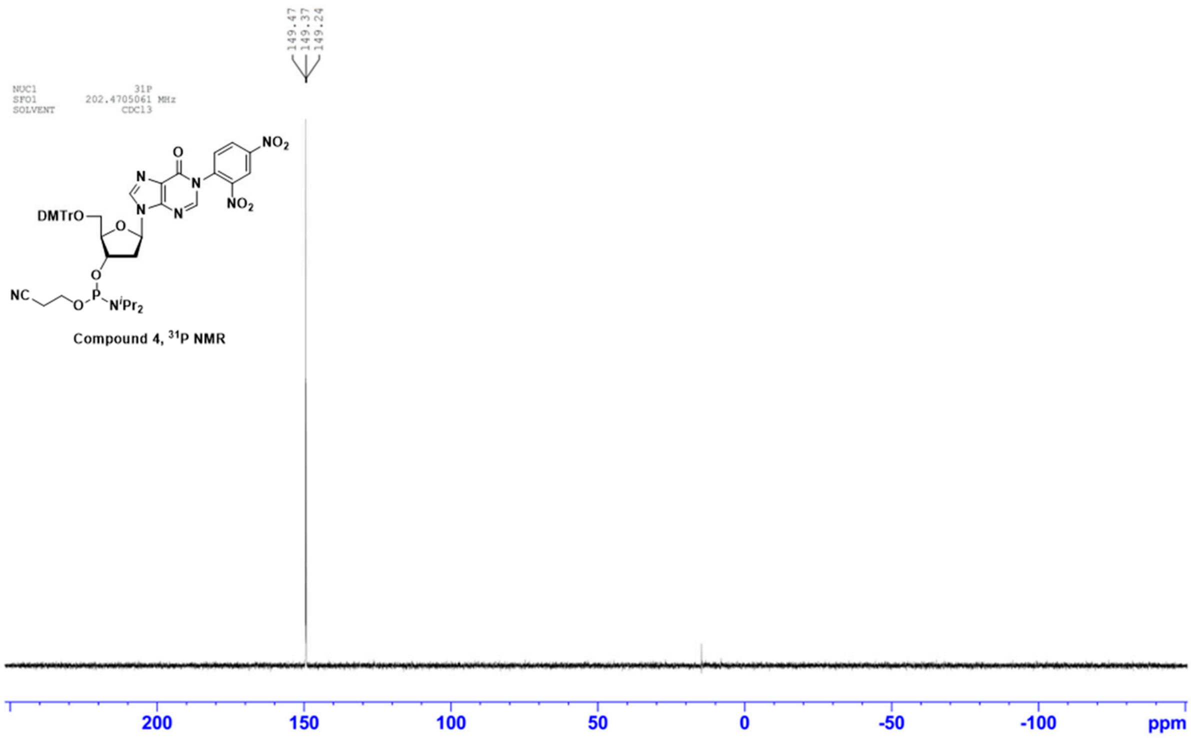
149.47
149.37
149.24
NUC1 31P
SFO1 202.4705061 MHz
SOLVENT CDCl3
Compound 4, 31P NMR
200
150
100
50
0
-50
-100
ppm

Cartesian coordinates of intermediates and transition states.

S1

Atom	x	y	z
N	4.64560800	0.01172800	-0.33724900
C	4.93334700	-0.95477800	0.60619900
H	5.95509300	-1.18382600	0.87862300
N	3.86688600	-1.53468700	1.10827400
C	2.81528900	-0.92058500	0.46894400
C	1.40041900	-1.13589000	0.62129400
O	0.79567500	-1.90991500	1.34087300
N	0.66672500	-0.23810200	-0.27147600
N	2.54767300	0.84490700	-1.24876900
C	3.27855800	0.03987700	-0.43011600
C	1.26784700	0.67010600	-1.12241100
H	0.58884500	1.25825400	-1.73343400
C	-0.75830000	-0.28682300	-0.20866000
C	-1.40974200	-1.47025300	-0.57624000
C	-1.54291200	0.81377500	0.17922400
C	-2.79731800	-1.55823500	-0.56862300
H	-0.80993800	-2.32420000	-0.86600500
C	-2.93464600	0.76013800	0.16117700
C	-3.53878300	-0.43457500	-0.20492300
H	-3.30587200	-2.47262800	-0.84761600
H	-3.52495400	1.62349700	0.43907600
N	-0.94379000	2.07911400	0.65949300
O	0.10752300	2.01294000	1.29109400

O	-1.56005100	3.11441600	0.41101500
N	-5.01428500	-0.50956700	-0.21126100
O	-5.52341800	-1.57680500	-0.54996900
O	-5.63600500	0.49822500	0.11976900
C	5.58239200	0.85013200	-1.07424700
H	5.43816000	0.72072500	-2.14934200
H	5.43243600	1.90213500	-0.81940600
H	6.59781700	0.55406700	-0.80695100

S2

N	-4.35030100	-1.46748900	-0.17763400
C	-4.25973000	-2.83919500	-0.31157100
H	-5.14055600	-3.45245400	-0.44973400
N	-3.02967000	-3.26784400	-0.25885400
C	-2.24547300	-2.13877900	-0.10451300
C	-0.78680300	-2.29322100	-0.00352800
O	-0.25388000	-3.37891700	0.17155600
N	-2.71983900	0.33785400	0.05283100
C	-3.05385600	-0.99832300	-0.03687400
C	1.32038300	-0.94717200	-0.07255500
C	2.15731800	-2.00396400	0.38733900
C	1.99017200	0.27558900	-0.41767200
C	3.52514100	-1.85960300	0.50089300
H	1.68832400	-2.94179100	0.64083400
C	3.37678500	0.41679300	-0.30053400
C	4.13230100	-0.64416300	0.15569300

H	4.13568400	-2.68011300	0.85781800
H	3.84450400	1.35314100	-0.57128000
N	1.29363800	1.44938600	-0.91972700
O	0.06165000	1.39161400	-1.07654200
O	1.94329500	2.46829000	-1.17059700
N	5.58332900	-0.48996300	0.27829200
O	6.22545100	-1.46183900	0.68354100
O	6.07722000	0.59766900	-0.02727200
C	-3.25484600	1.06228500	0.98491000
H	-3.87981100	0.63213800	1.77668500
N	-3.09932600	2.39652000	1.09114600
H	-3.53385000	2.83707700	1.88926600
C	-2.35416500	3.24847800	0.16371300
H	-2.04126900	2.62425200	-0.67337600
H	-3.01518600	4.03379200	-0.21919500
C	-1.13027100	3.89644100	0.82597500
H	-0.50428700	3.10160200	1.26439200
H	-1.46281900	4.53567200	1.65422700
N	-0.44476800	4.73699800	-0.15247400
H	0.20565300	5.37172800	0.29956200
H	0.09147500	4.16911200	-0.80481500
N	-0.04458000	-1.09376800	-0.15405200
H	-0.56545700	-0.25757100	-0.41857100
C	-5.56928200	-0.67941700	-0.30035100
H	-5.99307700	-0.42590600	0.67653000
H	-5.35987100	0.24016900	-0.85120900

H	-6.30477800	-1.26173200	-0.85884800

TS$_{S2-S3}$

N	-4.43481800	-1.88242800	0.27129200
C	-4.30729800	-2.82604700	-0.72521500
H	-5.12119100	-3.48984200	-0.98492100
N	-3.12555900	-2.81002500	-1.28148800
C	-2.42231000	-1.81148400	-0.63083100
C	-1.00738600	-1.57415300	-0.94061800
O	-0.46475300	-1.94807900	-1.98704900
C	-3.23604000	-1.20648400	0.34240800
C	1.05342900	-0.44471300	-0.09998700
C	1.31002300	0.36762800	-1.30703300
C	1.67357200	0.11376200	1.11551200
C	2.10265500	1.47147000	-1.29574500
H	0.82535000	0.04663900	-2.22028200
C	2.48414500	1.25250100	1.09488400
C	2.71001000	1.92656300	-0.08707100
H	2.26360400	2.04068200	-2.20386800
H	2.93298800	1.59408400	2.01880500
N	1.53556400	-0.57594400	2.35557300
O	0.99537000	-1.71532000	2.33682300
O	1.95962700	-0.06408400	3.39839100
N	3.54137200	3.10226200	-0.09747000
O	3.71069800	3.67968800	-1.18330500
O	4.04504000	3.48332900	0.96927600

C	-5.60608000	-1.62242700	1.09663200
H	-5.29112800	-1.46038200	2.12911800
H	-6.14705700	-0.73734100	0.74773500
H	-6.26792500	-2.48889900	1.04692700
C	3.93641700	-3.41519800	-0.88825000
H	3.68709300	-4.05139800	-0.02128900
H	3.47735800	-3.87909800	-1.76878800
C	3.33120100	-2.02050600	-0.68239200
H	3.50034800	-1.40663700	-1.57018500
H	3.81074900	-1.52072700	0.16618900
N	1.88433100	-2.06405700	-0.41716000
H	1.33194900	-2.41264400	-1.21033900
H	1.64903000	-2.58581300	0.43002200
N	5.37055700	-3.28428800	-1.14157900
H	5.88209200	-3.04939300	-0.29521700
H	5.76315800	-4.14469500	-1.50971700
N	-0.29511700	-0.90143100	0.04214300
H	-0.64283800	-0.97444800	0.99152400
N	-3.04543500	-0.25352900	1.32816500
C	-2.75386100	0.97661500	1.03694600
H	-2.59966900	1.65875500	1.87382100
N	-2.60779800	1.54646200	-0.17733100
H	-2.71938200	0.95233100	-0.99052800
C	-2.31012500	2.95815200	-0.39761100
H	-1.53229100	3.05111500	-1.16141100
H	-1.89458600	3.36426900	0.53145300

C	-3.54262100	3.76967100	-0.82395100
H	-3.94241100	3.34442800	-1.75208900
H	-4.32711100	3.65697600	-0.05607100
N	-3.14875000	5.15087800	-1.09307000
H	-3.87033700	5.65716900	-1.59553900
H	-2.95047300	5.66318300	-0.23803000

S3 (n=2)

N	1.73503500	-1.69281500	-0.17195300
C	3.09776000	-1.49394500	-0.24632100
H	3.78299000	-2.32358200	-0.36236500
N	3.42677800	-0.23136200	-0.16838300
C	2.23922700	0.46015400	-0.05325500
C	2.21805700	1.94430200	0.02960000
O	3.23795200	2.62545000	0.06136000
N	-0.19786200	-0.17660100	-0.00543600
C	1.16764000	-0.43161200	-0.04202200
C	-0.94830900	-0.80154400	0.83860900
H	-0.55870200	-1.49214500	1.59516900
N	-2.29654000	-0.66271300	0.86801300
H	-2.77512800	-1.03980300	1.67362500
C	-3.02886000	0.26220300	0.00902400
H	-2.89806400	1.29660200	0.36100100
H	-2.60655600	0.21079200	-0.99679200
C	-4.51619600	-0.09619000	-0.02260500
H	-4.89221200	-0.18377000	1.01440800

H	-4.63645300	-1.08007300	-0.48916000
N	-5.23849800	0.88339400	-0.83080900
H	-6.17989800	0.57041100	-1.04403500
H	-5.30933600	1.78172500	-0.36088400
N	0.96449700	2.51183400	0.08424100
H	0.14400000	1.95735800	-0.12611700
H	0.92720800	3.51501000	-0.02104500
C	1.05302700	-2.96664000	-0.34151500
H	0.77647400	-3.41663600	0.61790300
H	0.15263900	-2.82957700	-0.94516400
H	1.72147900	-3.65244600	-0.86622000

S3 (n=3)

N	2.12236800	-1.80981500	-0.08360800
C	3.46580100	-1.57264100	-0.25934800
H	4.17806700	-2.38154300	-0.35476300
N	3.74607900	-0.29513700	-0.30198000
C	2.53815000	0.35910500	-0.15157100
C	2.47763800	1.83960000	-0.23694900
O	3.41560800	2.56868900	0.06198400
N	0.13418900	-0.51188600	0.16937300
C	1.50848000	-0.57578400	0.00430400
C	-0.33561400	0.32025900	1.04168600
H	0.30241400	0.91253300	1.70449500
N	-1.66028500	0.53424700	1.20019600
H	-1.94773200	1.14904400	1.94666100

C	-2.68918800	-0.14006600	0.41559300
H	-2.39543700	-0.08614700	-0.63781800
H	-2.72221500	-1.20561900	0.68638900
C	-4.05583000	0.50972900	0.63727500
H	-4.01884100	1.55852900	0.31910500
H	-4.29597500	0.49092800	1.71014500
N	1.26631800	2.37071800	-0.65857300
H	0.64945900	1.80495000	-1.22540800
C	1.46684100	-3.09948600	0.05700700
H	1.16970900	-3.28075600	1.09484700
H	0.57547300	-3.13348900	-0.57313500
H	2.16326700	-3.87966000	-0.25643600
C	-5.18635600	-0.19573200	-0.12087700
H	-5.26385800	-1.23890600	0.23301200
H	-6.13081800	0.29420400	0.13776700
N	-5.01574300	-0.08206000	-1.57441700
H	-4.29883200	-0.71224800	-1.92255400
H	-5.87611900	-0.31169800	-2.06238300
H	1.29656200	3.35799400	-0.87546200

$TS_{S3\text{-}S4}$ (n=2)

N	-1.31920700	1.84587000	-0.05675700
C	-2.57069000	1.56712900	0.46135800
H	-3.26415800	2.35676900	0.72091400
N	-2.79497500	0.28718800	0.55769400
C	-1.65625000	-0.33605500	0.05914200

C	-1.61476300	-1.79484100	-0.05942100
O	-2.16218200	-2.58525900	0.70502100
C	-0.69100400	0.63116400	-0.30904700
N	0.59277900	0.65701000	-0.75172800
H	2.62892800	1.20685100	-1.12094400
C	1.38365900	-0.45991100	-0.62118400
H	1.34748600	-1.24930200	-1.39079600
C	3.60030200	0.19586700	0.47399400
C	2.45762500	-0.20331400	1.45064300
H	4.36484700	-0.58378600	0.43038600
H	4.07716200	1.14802200	0.71565900
H	2.83782700	-0.73930000	2.32260700
H	1.92306600	0.68488100	1.79573400
N	1.56356900	-1.04280500	0.67008600
H	1.85948900	-2.01400900	0.61964300
N	2.92883800	0.26507200	-0.85648900
H	3.47192900	-0.15875000	-1.60940900
N	-0.87690900	-2.28746100	-1.15968200
H	-1.06060600	-3.27398100	-1.30762200
H	-0.95693500	-1.73667300	-2.00694000
C	-0.72218300	3.14897900	-0.24691400
H	-1.44365700	3.91251300	0.05307300
H	0.18393400	3.25528500	0.36019800
H	-0.45069800	3.30532800	-1.29596000

TS$_{S3\text{-}S4}$ (n=3)

N	-0.93043800	2.00874200	-0.09373500
C	-2.11767900	2.03476400	0.61429000
H	-2.54598300	2.96448000	0.96698600
N	-2.63367000	0.84891200	0.76696500
C	-1.77469800	-0.02494500	0.10920800
C	-2.15535900	-1.42843700	-0.05567300
O	-2.93844700	-2.03584200	0.67028600
C	-0.66652600	0.68070300	-0.42172300
N	0.44530300	0.38566400	-1.13826300
H	2.87485400	0.36036000	-1.82269400
C	1.17270400	-0.75751200	-0.98909100
H	1.21863400	-1.41672700	-1.87270500
N	1.19386100	-1.60318000	0.18731700
H	0.36299600	-2.18887700	0.18196400
N	2.79638400	-0.24135800	-1.00265100
H	3.39216000	-1.06449100	-1.11469900
N	-1.53859600	-2.10509800	-1.13012600
H	-2.01902400	-2.97072200	-1.34741100
H	-1.32437800	-1.53881800	-1.94392800
C	-0.10566200	3.14442900	-0.44716300
H	-0.67879500	4.06025300	-0.28471000
H	0.80457600	3.19132000	0.16344200
H	0.18598700	3.08233100	-1.49866900
C	1.42829500	-0.91515500	1.46079500
H	1.30888700	-1.64886400	2.26263900

H	0.71086100	-0.10416800	1.65118000
C	2.86267300	-0.36978500	1.46669300
H	3.57227500	-1.20756200	1.47944700
H	3.04375400	0.22639800	2.36785200
C	3.12804700	0.51118300	0.24441800
H	2.47239700	1.38540400	0.23969200
H	4.16769900	0.84918000	0.19225100

S4 (n=2)

N	0.74357400	1.85555800	0.20304900
C	2.01571100	2.13926700	-0.21359100
H	2.34233000	3.15463500	-0.39484900
N	2.75556900	1.06229100	-0.35410800
C	1.93440400	0.01306700	-0.00639800
C	2.40391900	-1.39557800	-0.09008500
O	3.07659400	-1.83135000	-1.01446100
C	0.67057400	0.48238900	0.34601300
N	-0.53327200	-0.14824200	0.71334500
H	-0.37823900	-0.93801100	1.33000700
C	-1.38532300	-0.62811800	-0.39944900
H	-0.76299800	-1.04623800	-1.20286700
C	-3.67736900	-1.07915900	0.11260300
C	-3.43564100	0.42270500	-0.14362500
H	-4.27070200	-1.52054800	-0.70162000
H	-4.20502000	-1.25942600	1.05504500
H	-4.25274800	0.91038600	-0.68026800

H	-3.27869600	0.94938600	0.80308300
N	-2.18705800	0.47631100	-0.92578400
H	-2.38830900	0.29381700	-1.90684100
N	-2.30774600	-1.63074700	0.15335700
H	-2.23025500	-2.51512700	-0.33431800
N	1.96380200	-2.21884300	0.93912600
H	2.37147600	-3.14594200	0.93275700
H	1.84234600	-1.80880700	1.85548200
C	-0.31417500	2.81655700	0.48933000
H	-1.17571600	2.61961100	-0.15054900
H	-0.61782200	2.73678500	1.53677700
H	0.07491900	3.81919700	0.30215500

S4 (n=3)

N	-1.13176000	1.84359300	-0.24105400
C	-2.34592600	2.02617600	0.37553400
H	-2.71576300	3.01357300	0.61961500
N	-2.96972000	0.89671100	0.60486400
C	-2.15418400	-0.08891600	0.09260000
C	-2.56885900	-1.52024200	0.11353000
O	-3.55601500	-1.91931700	0.71952900
C	-0.99902000	0.47648400	-0.43878100
N	0.12068700	-0.12919800	-1.03266000
H	0.53623700	0.40489100	-1.79014900
C	1.19091800	-0.64860900	-0.14792000
H	0.76861300	-1.53203800	0.35851800

N	1.63004200	0.35930400	0.82567600
H	0.85334300	0.57883700	1.44435300
N	2.31933800	-1.05191000	-0.97091100
H	2.48132500	-2.04922900	-0.90725900
N	-1.75019300	-2.37452500	-0.59328700
H	-2.10875400	-3.30743300	-0.73863500
H	-1.07862000	-1.99511500	-1.25121000
C	-0.20554000	2.88176000	-0.67151400
H	0.81216500	2.60729500	-0.38389800
H	-0.25595100	3.03189100	-1.75581800
H	-0.47828700	3.81683900	-0.17880900
C	3.55703000	-0.28441800	-0.78470300
H	3.39410200	0.72718400	-1.16760500
H	4.32530100	-0.73954500	-1.41672900
C	4.01602500	-0.20973200	0.68783100
H	4.65615900	0.67027700	0.82294200
H	4.61803000	-1.08790800	0.94905800
C	2.77636500	-0.13487200	1.60761700
H	2.94382200	0.54980700	2.44352300
H	2.56677900	-1.12839900	2.04229600

Desired product S5

N	-1.70115800	-0.19516600	0.03815800
C	-1.32348700	-1.51955100	0.16771900
H	-2.05688900	-2.30779800	0.27277600
N	-0.02690200	-1.66922900	0.16664100

C	0.49762700	-0.39278500	0.06134600
C	1.95081800	-0.14811700	-0.03168500
O	2.75701800	-0.95385200	-0.47122800
C	-0.53728600	0.53569900	-0.02406400
N	-0.56451700	1.90291700	-0.29845100
H	-1.09422000	2.47092400	0.35434400
H	0.36578100	2.28027500	-0.44232800
C	-3.05016700	0.32972000	-0.08830400
H	-3.35910500	0.86624000	0.81595900
H	-3.10486800	1.00798200	-0.94445700
H	-3.73657600	-0.50332600	-0.24870800
N	2.35457100	1.13852000	0.36323900
H	1.90676000	1.51342900	1.19140700
H	3.36397500	1.23400100	0.37364700

$TS_{S3\text{-}S6}$ (n=2)

N	2.78093200	-0.56814100	0.25801400
C	3.09407500	0.61151500	0.89090900
H	4.07564900	0.78962500	1.30987000
N	2.10055800	1.46609300	0.89654300
C	1.07527700	0.81763600	0.22108300
O	-0.88141800	2.20039000	0.66126600
N	0.84437800	-1.57158900	-0.67861100
C	1.46490200	-0.47718100	-0.16953200
C	-0.47412400	-1.48918800	-0.82773500
H	-0.97767300	-2.44222700	-1.03773100

C	-2.64816600	-0.53610200	-0.59057900
H	-3.14335600	0.36273900	-0.98674600
H	-3.02837700	-1.38817300	-1.17514000
C	-3.06911900	-0.71673100	0.87646100
H	-2.67414900	-1.67330500	1.23780500
H	-2.60243600	0.07646300	1.47889400
N	-4.53437000	-0.76325500	0.97690300
H	-4.82596400	-1.08106200	1.89662600
H	-4.93538100	0.16183600	0.84248200
C	-0.18516400	1.49620300	-0.01436200
N	-1.20505200	-0.38672200	-0.74333400
H	-0.72913600	0.99106600	-2.00478100
N	-0.32296300	1.87629600	-1.65757600
H	-0.96448800	2.66347100	-1.76086500
H	0.58163500	2.08050200	-2.08194700
C	3.60570900	-1.76037900	0.15444300
H	4.60490100	-1.52630000	0.52739000
H	3.17547600	-2.57563100	0.74272500
H	3.66991600	-2.08554500	-0.88640300

$TS_{S3\text{-}S6}$ (n=3)

N	2.12236800	-1.80981500	-0.08360800
C	3.46580100	-1.57264100	-0.25934800
H	4.17806700	-2.38154300	-0.35476300
N	3.74607900	-0.29513700	-0.30198000
C	2.53815000	0.35910500	-0.15157100

C	2.47763800	1.83960000	-0.23694900
O	3.41560800	2.56868900	0.06198400
N	0.13418900	-0.51188600	0.16937300
C	1.50848000	-0.57578400	0.00430400
C	-0.33561400	0.32025900	1.04168600
H	0.30241400	0.91253300	1.70449500
N	-1.66028500	0.53424700	1.20019600
H	-1.94773200	1.14904400	1.94666100
C	-2.68918800	-0.14006600	0.41559300
H	-2.39543700	-0.08614700	-0.63781800
H	-2.72221500	-1.20561900	0.68638900
C	-4.05583000	0.50972900	0.63727500
H	-4.01884100	1.55852900	0.31910500
H	-4.29597500	0.49092800	1.71014500
N	1.26631800	2.37071800	-0.65857300
H	0.64945900	1.80495000	-1.22540800
C	1.46684100	-3.09948600	0.05700700
H	1.16970900	-3.28075600	1.09484700
H	0.57547300	-3.13348900	-0.57313500
H	2.16326700	-3.87966000	-0.25643600
C	-5.18635600	-0.19573200	-0.12087700
H	-5.26385800	-1.23890600	0.23301200
H	-6.13081800	0.29420400	0.13776700
N	-5.01574300	-0.08206000	-1.57441700
H	-4.29883200	-0.71224800	-1.92255400
H	-5.87611900	-0.31169800	-2.06238300

H	1.29656200	3.35799400	-0.87546200

By-product S6 (n=2)

N	2.87503800	-0.22311700	0.16119000
C	3.03651500	1.14597200	0.24723400
H	4.01297000	1.58436300	0.40631900
N	1.91601600	1.81832400	0.11599000
C	0.95630700	0.84712800	-0.06416500
C	-0.46622800	0.97563300	-0.25919800
O	-1.15634400	1.98547300	-0.30782400
N	-1.07388900	-0.32550100	-0.40789000
N	0.91236700	-1.62455000	-0.18441700
C	1.53153200	-0.42379700	-0.03814000
C	-0.37572600	-1.50193500	-0.36381700
H	-0.97147600	-2.40074200	-0.49806700
C	-2.53204300	-0.35776700	-0.60773100
H	-2.78911400	0.49872300	-1.23488500
H	-2.79239000	-1.27296600	-1.14486700
C	-3.30601400	-0.28513000	0.71448400
H	-3.09867900	-1.18673100	1.30283500
H	-2.94426900	0.57898900	1.28985100
N	-4.74125300	-0.25360600	0.41900900
H	-5.29106400	-0.42520900	1.25520700
H	-5.01639600	0.65726000	0.05978000
C	3.89643200	-1.25369500	0.27167600
H	3.72095600	-1.87439300	1.15433400

H	3.88648700	-1.89148900	-0.61537400
H	4.87112600	-0.77089900	0.35822300

By-product S6 (n=3)

N	3.26851400	-0.30210400	0.30249700
C	3.46983500	1.05375500	0.47321200
H	4.44226500	1.44451900	0.74291800
N	2.39057800	1.77550600	0.27581600
C	1.41788500	0.85342800	-0.04110400
C	0.02459000	1.04948400	-0.35650900
O	-0.62022100	2.08745600	-0.41484700
N	-0.61260900	-0.21845600	-0.62703500
N	1.29783200	-1.60528200	-0.29396800
C	1.94308800	-0.43894600	-0.02896800
C	0.03665700	-1.42160100	-0.58117400
H	-0.57529900	-2.28911000	-0.81367600
C	-2.05308400	-0.18598000	-0.93800400
H	-2.23261000	0.73434900	-1.49774600
H	-2.26994900	-1.03662800	-1.59207600
C	-2.92823700	-0.22124400	0.31961900
H	-2.72925000	-1.14958100	0.87428900
H	-2.66235100	0.61301500	0.97544900
C	4.23737800	-1.37700400	0.45738300
H	3.97906500	-2.00739700	1.31269300
H	4.25875700	-1.99529400	-0.44303500
H	5.22341600	-0.93764900	0.61748400

C	-4.41969200	-0.13722600	-0.01808700
H	-4.67339500	-0.91981900	-0.75658800
H	-4.62477200	0.82745200	-0.49695100
N	-5.22560800	-0.20517400	1.20470500
H	-6.20485500	-0.01652500	1.01203300
H	-5.17073400	-1.12640200	1.63167700
